# Treatment of Yellow Fever

**Published:** 1889-11-16

**Authors:** 


### TREATMENT OF YELLOW FEVER.

In a recent number of the *Philadelphia Therapeutic Gazette*, Major and Surgeon George M. Sternberg, U.S.A., publishes some observations on bicarbonate of sodium and bichloride of mercury in the treatment of yellow fever. The formula used was: "Sodæ bicarb., grs. 150; hydrog. chloridi corrosiv $\frac{3}{10}$ gr.; aquæ, 1 quart; about $1\frac{3}{4}$ oz. every hour, to be given ice cold." The soda was prescribed with the idea of neutralising the very acid condition of the urine. Bichloride of mercury was used not so much with the idea that it would destroy micro-organisms, but as an antiseptic which might be useful in preventing fermentative changes. From statistics given it would appear that this treatment was very successful. Fortunately, we do not see yellow fever in this climate. But it will be well for those visiting the West, where yellow fever prevails, to make a note of Surgeon and Major Sternberg's treatment.

